# P59 Linezolid in short- term regimens for drug-resistant TB: balancing efficacy and toxicity

**DOI:** 10.1093/jacamr/dlaf230.066

**Published:** 2025-12-04

**Authors:** Anna Abramchenko, Anastasia Gaida, Mariya Romanova, Galina Mozhokina, Irina Burmistrova, Anastasia Samoilova, Irina Vasilyeva

**Affiliations:** Federal State Budgetary Institution ‘National Medical Research Center for Phthisiopulmonology and Infectious Diseases’ of the Ministry of Health of the Russian Federation, Moscow, Russia; Pirogov Russian National Research Medical University, Moscow, Russia; Pirogov Russian National Research Medical University, Moscow, Russia; Pirogov Russian National Research Medical University, Moscow, Russia; Pirogov Russian National Research Medical University, Moscow, Russia; Federal State Budgetary Institution ‘National Medical Research Center for Phthisiopulmonology and Infectious Diseases’ of the Ministry of Health of the Russian Federation, Moscow, Russia; Pirogov Russian National Research Medical University, Moscow, Russia; Pirogov Russian National Research Medical University, Moscow, Russia; Federal State Budgetary Institution ‘National Medical Research Center for Phthisiopulmonology and Infectious Diseases’ of the Ministry of Health of the Russian Federation, Moscow, Russia; Pirogov Russian National Research Medical University, Moscow, Russia

## Abstract

**Background:**

Short-term regimens with linezolid are an effective treatment for patients with MDR and pre-XDR TB (MDR/pre-XDR TB). However, their use is limited by the risk of adverse drug reactions (ADRs) associated with this drug.

**Objective:**

To analyse the efficacy and risk of ADRs in patients with MDR/pre-XDR TB undergoing short-term regimens that include linezolid.

**Patients and methods:**

The study included 61 patients with MDR/pre-XDR TB who received 9 month treatment regimens between 2023 and 2024 from five regions of the Russian Federation. The five-drug combination included bedaquiline, linezolid, delamanid, cycloserine/terizidone and clofazimine/levofloxacin/moxifloxacin/pyrazinamide. Among them, 34 (55.7%) were male and 27 (44.3%) were female. The median age was 40.0 [32.0–46.5] years. The study included 54 (88.5%) patients with newly diagnosed TB and 7 (11.5%) with disease relapse. Laboratory confirmation of the diagnosis by rapid TB tests was registered in 58 patients (95.1%), and drug resistance was confirmed in all by culture-based testing. MDR-TB was registered in 45 (73.8%) and pre-XDR TB in 16 (26.2%) patients. Patients had no concomitant pathologies in the decompensation stage. Diabetes was present in 3 (4.9%) patients and hepatitis C in 4 (6.6%). The severity of ADRs was assessed using the CTCAE scale.

**Results:**

Cessation of bacterial excretion was observed in all patients by the 4th month of treatment; however, the treatment course was successfully completed by 58 patients (95.1%). A total of 52 ADRs were registered in 28 (45.9%) patients. The most common were polyneuropathy (13 cases; 25.0%), diarrhoea (7 cases; 13.5%), nausea and myelosuppression (6 cases each; 11.5% each). Grade 1–2 ADRs occurred in 46 cases (88.5%). Grade 3 and higher ADRs occurred in 6 cases (11.5%) (leukopenia, neutropenia and erythrocytopenia (one case each) and polyneuropathy (3 cases; 50.0%)). Linezolid was the causative drug in 36.5% of all ADRs (in 22 patients). Due to the development of ADRs, therapy was modified for eight patients: complete discontinuation of linezolid was required for four patients, and a dose reduction from 600 mg to 300 mg was required for another four.

**Conclusions:**

Short-term regimens including linezolid are characterized by high treatment efficacy (95.1%) but a high frequency of associated ADRs (36.5% of patients). This underscores the need for careful monitoring of ADRs for timely treatment adjustment.

